# Blockage of Autophagic Flux and Induction of Mitochondria Fragmentation by Paroxetine Hydrochloride in Lung Cancer Cells Promotes Apoptosis via the ROS-MAPK Pathway

**DOI:** 10.3389/fcell.2019.00397

**Published:** 2020-01-22

**Authors:** Kun Wang, Qing Gong, Yujuan Zhan, Bonan Chen, Ting Yin, Yuhua Lu, Yilin Zhang, Huiqi Wang, Junzi Ke, Biaoyan Du, Xiaodong Liu, Jianyong Xiao

**Affiliations:** ^1^Research Center of Integrative Medicine, School of Basic Medical Sciences, Guangzhou University of Chinese Medicine, Guangzhou, China; ^2^Department of Pathology, Guangzhou University of Chinese Medicine, Guangzhou, China; ^3^GMU-GIBH Joint School of Life Sciences, Guangzhou Medical University, Guangzhou, China; ^4^Department of Biochemistry, Guangzhou University of Chinese Medicine, Guangzhou, China; ^5^Department of Anaesthesia and Intensive Care, The Chinese University of Hong Kong, Shatin, Hong Kong

**Keywords:** paroxetine hydrochloride, lung cancer, autophagy, ROS, apoptosis

## Abstract

Cancer cells are characterized by malignant proliferation and aberrant metabolism and are thereby liable to the depletion of nutrients and accumulation of metabolic waste. To maintain cellular homeostasis, cancer cells are prone to upregulating the canonical autophagy pathway. Here, we identified paroxetine hydrochloride (Paxil) as a late autophagy inhibitor and investigated its killing effect on lung cancer cells and with a xenograft mouse model *in vivo*. Upregulated LC3-II and p62 expression indicated that Paxil inhibited autophagy. Acid-sensitive dyes (e.g., LysoTracker and AO staining) indicated reduced lysosomal acidity following Paxil treatment; consequently, the maturation of the pH-dependent hydroxylases (e.g., cathepsin B and D) substantially declined. Paxil also induced the fragmentation of mitochondria and further intensified ROS overproduction. Since the autophagy pathway was blocked, ROS rapidly accumulated, which activated JNK and p38 kinase. Such activity promoted the localization of Bax, which led to increased mitochondrial outer membrane permeability. The release of Cytochrome c with the loss of the membrane potential triggered a caspase cascade, ultimately leading to apoptosis. In contrast, the clearance of ROS by its scavenger, NAC, rescued Paxil-induced apoptosis accompanied by reduced p38 and JNK activation. Thus, Paxil blocked the autophagic flux and induced the mitochondria-dependent apoptosis via the ROS-MAPK pathway.

## Introduction

Autophagy is a highly conserved degradative process through which cells can recycle intracellular waste products to maintain cellular homeostasis (White, 2012). For cancer cells, malignant proliferation and abnormal metabolism leave cells depleted of nutrients and full of stressful waste products (e.g., dysfunctional mitochondria and its byproduct, reactive oxygen species [ROS]). Theoretically, elevated levels of autophagy may protect cancer cells from programed cell death and thereby exhibit tumor-facilitating activity (White, 2012). Indeed, a high level of autophagy was observed in the tumor cells and believed to be a cell survival process that affected multiple processes, including anoikis, cell motility, and EMT (Mowers et al., 2017). Thus, the blockage of autophagic flux might be beneficial for the treatment of various cancers.

The blockage of autophagy may result in the accumulation of intracellular ROS, including peroxides, superoxides, and hydroxyl radicals. Compared with normal cells, cancer cells have a dominant number of dysfunctional mitochondria due to frequent fission and fusion events. Damaged mitochondria are the major sources of high levels of ROS production (Indo et al., 2007). Thus, with the blockage of the autophagy pathway, higher levels of ROS can possibly accumulate to a greater extent in cancer cells compared to normal cells. In most cases, excessive ROS is detrimental to the cells. For instance, ROS may activate JNK, leading to Bcl-2 or p53-dependent mitochondrial apoptosis (Shen and Liu, 2006; Shi et al., 2014; Redza-Dutordoir and verill-Bates, 2016); p38 MAPK can also be activated by ROS and cause apoptosis (Liu et al., 2014; Wu et al., 2017). Therefore, the present study sought to determine whether the inhibition of autophagy could preferentially kill cancer cells over normal cells. Collectively, autophagy inhibitors may cause the accumulation of ROS and exert cytotoxic activity toward cancer cells.

Lung cancer is the leading cause of cancer-related death globally, accounting for 18.4% of death to all cancers in 2018 (Bray et al., 2018). Despite therapeutic advances (e.g., radiation, chemotherapy, and targeted drugs), intolerable adverse effects and drug resistance is limited to the efficacy of these regimens. Novel compounds with anti-cancer activity, especially medications that have been regularly prescribed for non-cancer diseases, have the potential to become valuable alternatives to current therapies. We performed a preliminary screening of many small molecules provided by Gu et al. (2013) and discovered that Paxil exhibited considerable cancer suppressive capacity accompanied by its functions as an autophagy inhibitor. Paroxetine hydrochloride (Paxil), a phenylpiperidine derivative, is a selective serotonin reuptake inhibitor prescribed to treat mood disorders (e.g., depression, generalized anxiety disorders, and obsessive-compulsive disorder) (Germann et al., 2013). Based on the excellent medicinal characteristics of Paxil, in the present study, we sought to evaluate its anti-cancer activity both *in vitro* and *in vivo*, and further elucidate the associated underlying mechanisms in lung cancer cells.

## Results

### Paxil Inhibits the Proliferation of NSCLC Cells

**FIGURE 1:**
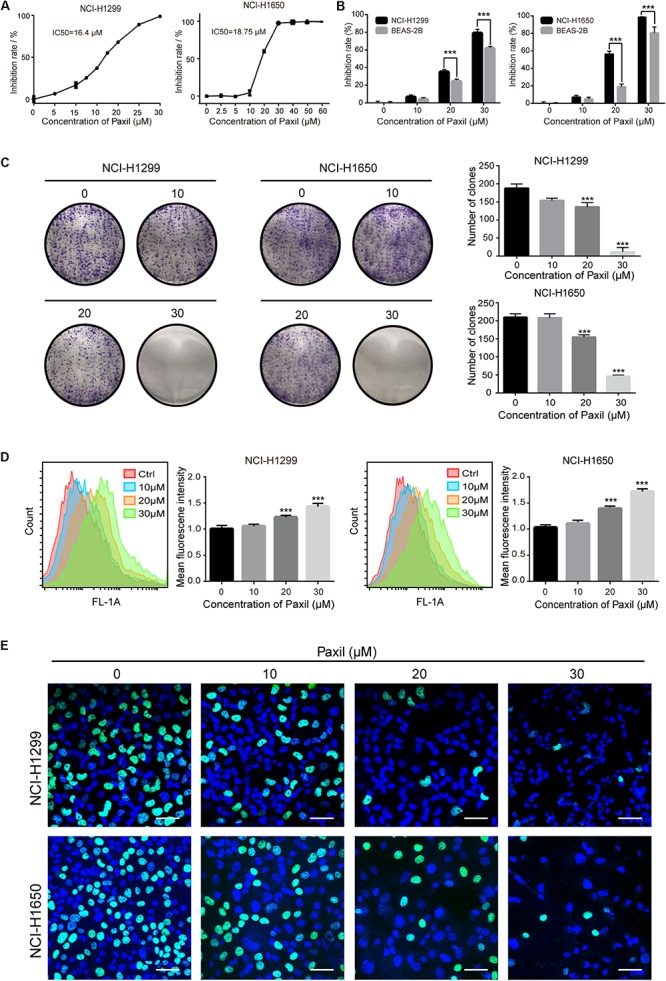
Paxil inhibited the proliferation of human NSCLC cells. **(A)** NCI-H1299 and NCI-H1650 cells were treated with a gradient concentration of Paxil for 24 h, and cell viability was determined with a CCK8 assay and visualized with GraphPad software. The median inhibitory concentration (IC50) was estimated by log (inhibitor) vs. normalized response non-linear fit. **(B)** Inhibition rate of NSCLC cells vs. normal human lung epithelial cells (BEAS-2B) following Paxil treatment for 24 h determined by a CCK8 assay. **(C)** Paxil inhibited the colony formation of NSCLC cells. Cells were exposed to Paxil for seven days and then stained by crystal violet. **(D)** Cells were labeled with CFDA-SE and treated with Paxil for 24 h. The mean fluorescence intensity was inversely correlated with cell division was determined by flow cytometry. **(E)** Cells were treated with Paxil for 24 h. EdU-positive cells were marked in green. Blue represents nuclei labeled with Hoechst 33342. Images were obtained by a confocal laser scanning microscope. Scale bar: 20 μm. Error bars, means ± SD of three independent experiments; ^∗∗∗^*p* < 0.001, compared to the control.

We first sought to determine whether Paxil had a potential anti-cancer effect on NSCLC using CCK8 assay. The data in Figure 1A showed that Paxil inhibited the cell viability of NSCLC cells in a dose-dependent fashion compared to vehicle control. The IC50 of Paxil on NCI-H1299 and NCI-H1650 cells was 16.4 and 18.75 μM, respectively. Intriguingly, Paxil induced a higher inhibition rate for NSCLC compared to a normal human lung epithelial cell line (BEAS-2B), which suggests that Paxil might preferentially kill cancer cells compared to normal cells (Figure 1B). To evaluate the cytotoxicity of Paxil, we also examined the cell viability of various types of cancer cells, and the IC50 of Paxil on A375, HepG2, MCF-7, and CNE1 cells for 24 h was 13.99, 15.86, 17.38, and 18.74 μM, respectively (Supplementary Figure S1). The inhibitory effect of Paxil on lung cancer cells was also confirmed by a colony formation assay. There was a reduced number of colonies that formed in the Paxil group compared with vehicle control (Figure 1C). At a concentration of 30 μM, Paxil completely blocked the colony-forming capability of both NCI-H1299 and NCI-H1650 cells (Figure 1C). Moreover, using CFDA-SE staining, we also monitored the proliferation rate of NSCLC following Paxil treatment. CFDA-SE is a fluorescent dye that is evenly distributed among dividing cells and the mean intensity of fluorescence decreases with each division. In contrast, in non-dividing cells, the fluorescence is sequestered, and the intensity is not attenuated. As demonstrated in Figure 1D, the mean fluorescent intensity in NSCLC cells was significantly higher in the Paxil group compared to that in the vehicle group, indicating that fewer cells underwent division following Paxil treatment. Compared to the vehicle, Paxil treatment also significantly reduced the incorporation of 5-ethynyl-20-deoxyuridine (EdU) into DNA, compared to vehicle control (Figure 1E). This result was in agreement with the CFDA-SE assay and demonstrated that DNA replication was inhibited by Paxil in a dose-dependent manner. Taken together, these data suggest that Paxil represents a novel inhibitor of NSCLC cell proliferation.

### Paxil-Induces Damage of Mitochondria and Simultaneously Inhibits Autophagy in NSCLC Cells

**FIGURE 2:**
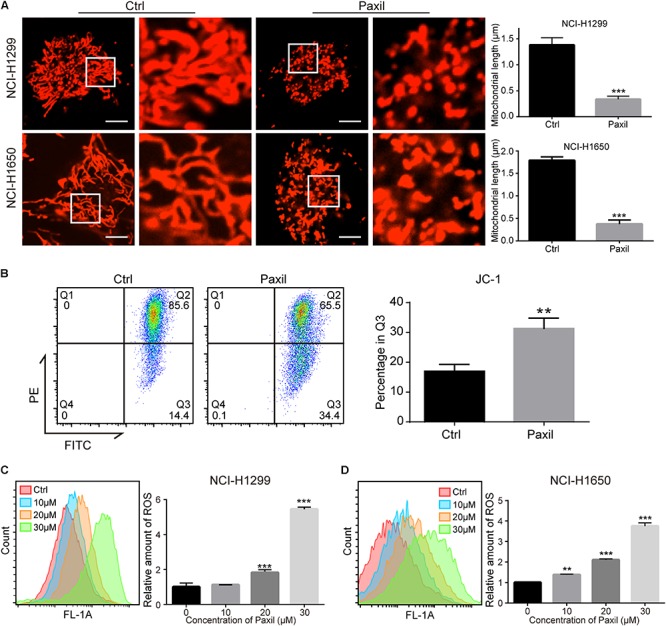
Paxil induced fragmentation of mitochondria in NSCLC cells. **(A)** NCI-H1299 and NCI-H1650 cells were treated with either a vehicle or Paxil (20 μM) for 24 h. The mitochondria were stained by MitoTracker Red CMXRos. The length of the mitochondria was quantified in ImageJ. Scale bar: 5 μm; ^∗∗∗^*p* < 0.001. **(B)** Paxil treatment caused a significant decrease of mitochondrial membrane potential (MMP). NCI-H1299 cells were treated with either a vehicle or Paxil (20 μM) for 24 h. The MMP was determined by JC-1 assay. ^∗∗^*p* < 0.01. **(C,D)** Cells were treated with Paxil at the indicated concentrations and dyed with the green fluorescent dye DCFDA for labeling intracellular ROS. The green mean fluorescence intensity that indicated the relative amount of ROS was detected by flow cytometry. ^∗∗^*p* < 0.01; ^∗∗∗^*p* < 0.001.

Cellular viability or proliferation has a tight relationship with mitochondrial function. We observed the morphology of mitochondria stained with MitoTracker Red. The data showed a dramatic reduction in mitochondrial length following Paxil treatment (Figure 2A), which was indicative of fragmented mitochondria. Then we performed JC-1 assay to determine the effect of Paxil on mitochondrial membrane potential (MMP). As shown in Figure 2B, Paxil treatment caused a significant decrease of MMP in NCI-H1299 cells. The intracellular level of ROS as a byproduct of mitochondrial damage is likely to become elevated with an increase in damaged mitochondria. Indeed, Paxil upregulated the level of ROS in a dose-dependent manner in both cell lines, with as high as a four-fold elevation at the highest concentration (Figures 2C,D).

**FIGURE 3:**
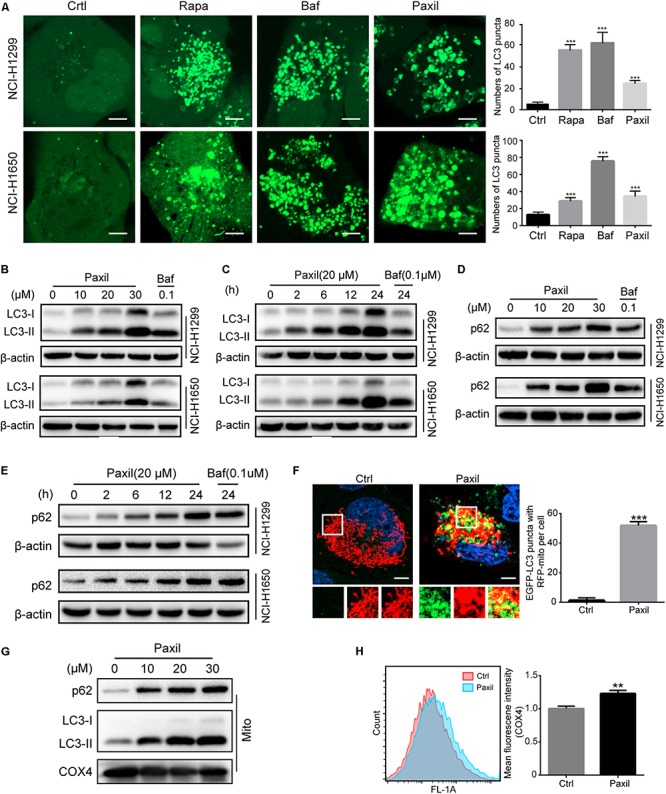
Paxil inhibited autophagy in NSCLC cells. **(A)** The increased GFP-LC3 puncta with Paxil treatment. NSCLC cell lines (NCI-H1299 and NCI-H1650) that stably over-express GFP-LC3 were treated with a vehicle, rapamycin (Rapa, 0.5 μM), bafilomycin A1 (Baf, 0.1 μM), and Paxil (20 μM) for 24 h. Images were obtained with a confocal laser scanning microscope. Scale bar: 5 μm. **(B–E)** Cells were treated with Paxil or Baf at the concentration gradient for 24 h or at the indicated dose over a time course. The protein level was examined by western blot. **(F)** Colocalization of mitochondria and autophagosomes. An NCI-H1299^GFP–LC3^ stable cell line was transiently transfected with a pDsRed_2_-Mito plasmid and treated with either a vehicle or Paxil (20 μM) for 24 h. Green represents the autophagosomes labeled with GFP-LC3; red indicates the mitochondria labeled Red_2_-Mito. Representative images are shown. Scale bar: 5 μm; ^∗∗∗^*p* < 0.001. **(G)** The upregulation of p62 and LC3-II in mitochondrial fractions induced by Paxil. Following Paxil treatment for 24 h, the mitochondrial parts in NCI-H1299 cells were separately extracted from the total lysates. The protein level was examined by western blot. COX4 was used as an internal reference for the mitochondrial fractions. Mito, mitochondrial fractions. **(H)** NCI-H1299 cells were treated with either a vehicle or Paxil (20 μM) for 24 h, after which the cells were harvested and stained with an immunofluorescence assay. The mean fluorescence intensity of COX4, a mitochondrial marker, was detected by flow cytometry.

To maintain redox homeostasis and avert the detriment of ROS, cancer cells usually adapt to recycle the damaged mitochondria through the induction of autophagy (Ashrafi and Schwarz, 2013). Indeed, there was a substantial increase in the number of autophagosomes in NSCLC cells with exposure to Paxil, as evidenced by an elevated level of LC3-II (an autophagosome marker). As shown in Figure 3A, Paxil induced the accumulation of green fluorescence in NSCLC cells stably overexpressing GFP-LC3. In addition, the immunoblotting results showed that compared to the vehicle control, the ratios of LC3-II/LC3-I and LC3-II/β-actin were gradually increased with exposure to Paxil in a dose- and time-dependent manner (Figures 3B,C). Of note, the increase in autophagosomes may result from either the induction of autophagy using Rapamycin (Rapa, 0.5 μM) or the blockage of the late autophagic flux, similar to that achieved following treatment with Bafilomycin A1 (Baf, 0.1 μM). Thus, the level of p62/SQSTM1 protein expression was assessed to differentiate autophagy induction or autophagic flux impairment. As demonstrated in Figure 3D, Paxil treatment induced a striking increase of p62 protein in both cell lines in a dose-dependent manner. With a concentration of 20 μM, Paxil induced p62 elevation as early as 2 h, which persisted for at least 24 h in both cell lines (Figure 3E). Since p62 is degraded through autophagy, its upregulation represented a blockage of the autophagy pathway. Compared with Baf, a canonical inhibitor of autophagy, the modulation of LC3-II and p62 levels by Paxil implied that Paxil was a potent autophagy inhibitor.

These data suggest that Paxil induces mitochondrial damage in lung cancer cells. Such damaged mitochondria can be removed via the autophagy pathway. As demonstrated above, Paxil impaired autophagy flux by affecting the late stage of the autophagy pathway. Therefore, it is possible that mitophagy can be initialized but is arrested during the clearance stage. Compared with vehicle control, GFP-LC3 puncta were significantly induced and co-localized with mitochondria (red) after Paxil treatment (Figure 3F). The upregulation of LC3-II and p62 in the separated mitochondria following Paxil treatment corroborated the conclusion that mitophagy-associated compartments were formed and engulfed damaged mitochondria following Paxil treatment (Figure 3G). COX4, a mitochondrial marker, was quantified to determine the mitochondrial mass via flow cytometry. Although mitophagy was initialized, the mitochondrial mass was significantly increased in Paxil-treated cells compared with the vehicle-treated cells (Figure 3H).

### Damaged Mitochondria Pack Into Autophagosomes Relying on a Ubiquitin Pathway

**FIGURE 4:**
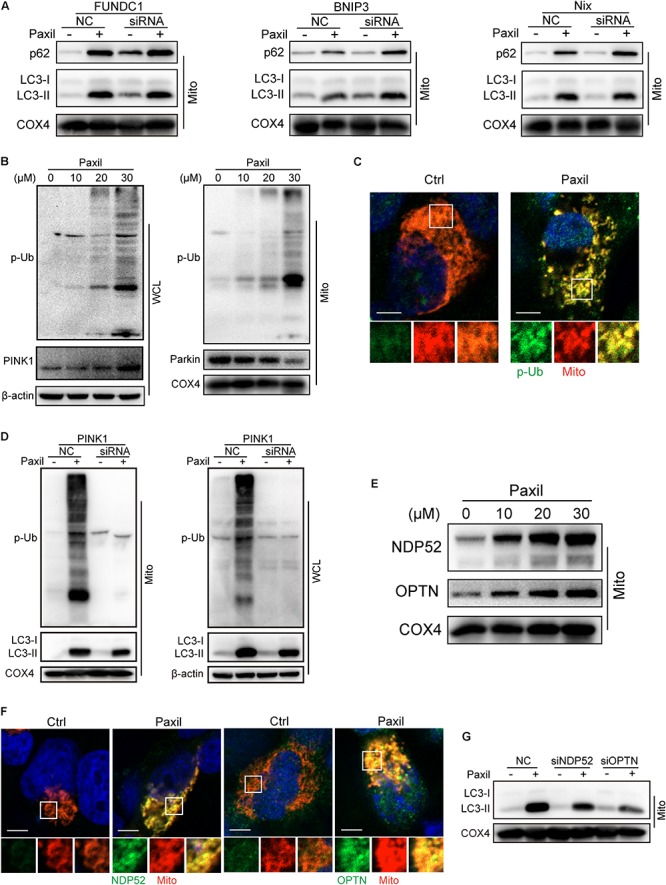
Autophagosomes recognized and bound to the impaired mitochondria possibly relying on the ubiquitination process rather than receptor-mediated pathways. The mitochondrial parts in NCI-H1299 cells were separately extracted from the total lysates. The protein level was examined by western blot. **(A)** The expression of LC3-II and p62 in lung caner cells with the silence of FUNDC1, BNIP3 and Nix. NCI-H1299 cells were transfected with small interfering RNA and cultured for 24 h, subsequently treated with Paxil as indicated. **(B)** NCI-H1299 cells were treated with Paxil for 24 h, then the indicated proteins were detected by western blot assay. **(C)** Co-localization of mitochondria and phosphorylated ubiquitin. The mitochondria were marked as red by pDsRed2-mito plasmids transfection, then immunofluorescence experiments were performed to stain phosphorylated ubiquitin (p-Ub) as green. Scale bar, 5 μm. **(D)** PINK1 knockdown blocked mitophagy induced by Paxil treatment. NCI-H1299 cells were transfected with small interfering RNA and cultured for 24 h, subsequently treated with Paxil as indicated. Then the indicated proteins were detected by western blot assay. **(E)** The upregulation of Optineurin and NDP52 proteins in the mitochondrial fractions induced by Paxil. COX4 was used as an internal reference for the mitochondrial fractions. **(F)** Co-localization of mitochondria and NDP52 or OPTN. The mitochondria were marked as red by pDsRed2-mito plasmids transfection, then immunofluorescence experiments were performed to stain NDP52 or OPTN as green. Scale bar, 5 μm. **(G)** Either NDP52 or OPTN knockdown reversed Paxil-induced mitophagy. NCI-H1299 cells were transfected with small interfering RNA and cultured for 24 h, subsequently treated with Paxil as indicated. Then the indicated proteins were detected by western blot assay. WCL, whole-cell lysates; Mito, mitochondrial fractions.

Now that the damaged mitochondria can be wrapped into the autophagosomes after Paxil treatment, next we dug into the mechanisms underly this process. Mitophagy was usually intermediated by ubiquitin-dependent pathways or receptor-mediated pathways (Harris et al., 2017). Knockdown of several major receptors localized in the mitochondria such as FUNDC1, BNIP3 and Nix by small interfering RNA did not reduce the extent of mitophagy, suggesting that the receptors are unlikely involved in mitophagy (Figure 4A).

In contrast, the elevated expression of PINK1 and phosphorylated ubiquitin (p-Ub) suggested that ubiqutination might be the mechanism that mediated the Paxil-induced mitophagy (Figure 4B). We also determined the co-localization of mitochondria and p-Ub. As shown in Figure 4C, p-Ub was mainly distributed on the mitochondria. Parkin, a key ubiquitin ligase in the ubiquitin-mediated mitophagy, however, seems not to participate in the ubiquitination process due to the downregulation of protein expression (Figure 4B). In order to prove the necessity of PINK1 for Paxil-induced mitophagy, we suppressed the expression of PINK1 by a small interfering RNA, and found that p-Ub and LC3-II upregulation induced by Paxil was indeed reversed (Figure 4D). Nonetheless, in the absence of Parkin, the basal ubiquitin of outer mitochondrial membrane followed by the phosphorylation by PINK1 is reported to be sufficient to mediate the mitophagy process, wherein Optineurin (OPTN) and NDP52 can be recruited to mitochondria (Stolz et al., 2014; Wong and Holzbaur, 2014; Michael, 2015), and bind to the phosphorylated basal ubiquitin (Nguyen et al., 2016). NDP52 and OPTN are important autophagy mediators which are thought to function primarily by bridging LC3 and ubiquitinated cargo (Svenning and Johansen, 2013; Stolz et al., 2014; Lazarou et al., 2015). In agreement with these reports, the protein level of OPTN and NDP52 did increase after Paxil induction in the mitochondrial fractions (Figure 4E) and both OPTN and NDP52 co-localized with LC3 (Supplementary Figure S2). Figure 4F showed that NDP52 and OPTN were also mainly distributed on the mitochondria which was similar to the distribution of p-Ub. Then we conducted small interfering RNA transfection assay to confirm the necessity of NDP52 and OPTN for Paxil-induced mitophagy and the data demonstrated that either NDP52 or OPTN knockdown could block Paxil-induced mitophagy (Figure 4G). Collectively, mitophagy induced by Paxil might be mediated through a ubiquitination process during which PINK1, NDP52, OPTN played important roles.

### Paxil Blocks Autophagic Flux by Inhibiting Lysosomal Acidification Rather Than Interfering With Autophagosome and Lysosome Fusion

**FIGURE 5:**
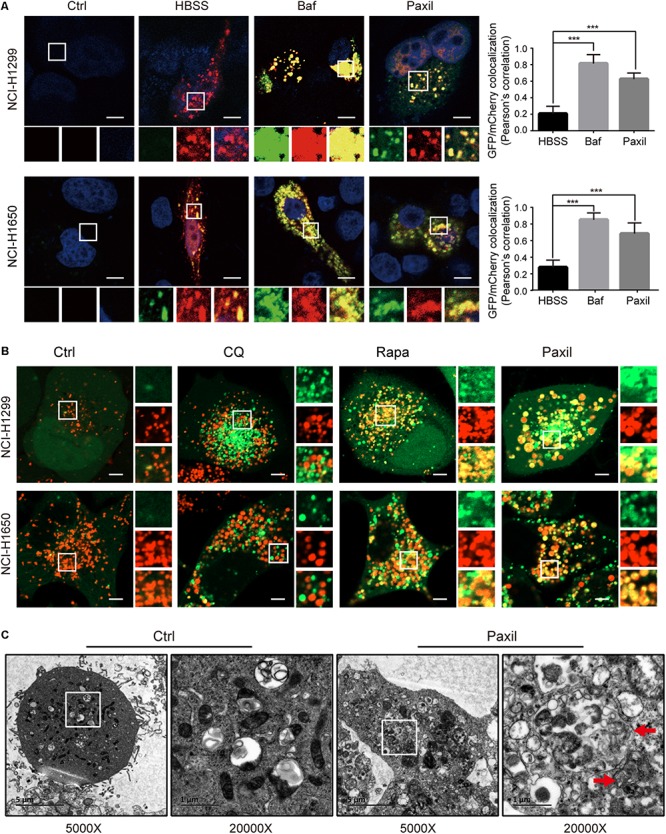
Paxil did not block autophagic flux by interfering with autophagosome and lysosome fusion. **(A)** NCI-H1299 and NCI-H1650 cells were transiently transfected with mCherry-GFP-LC3 plasmids and treated with a vehicle, Paxil (20 μM), Baf (0.1 μM) for 24 h, or HBSS for 6 h. Representative images are shown. Scale bar: 5 μm. Error bars represent the means ± SD of three independent experiments; ^∗∗∗^*p* < 0.001, compared to the control. **(B)** NCI-H1299 and NCI-H1650 cells stably expressing GFP-LC3, a canonical autophagosome marker with green fluorescence, were treated with a vehicle, chloroquine (CQ, 20 μM), Rapamycin (Rapa, 0.5 μM), or Paxil (20 μM) for 24 h. LysoBrite red dyes were used to label the lysosomes. Images were acquired with a confocal laser scanning microscope. Typical images are shown. Scale bar: 5 μm. **(C)** NCI-H1299 cells were treated by a vehicle or Paxil (20 μM) for 24 h. A transmission electron microscopy (TEM) assay was performed to observe the cellular structures. In each group, the image on the right is a partial enlargement of the image on the left. Red arrow, autolysosomes.

To further monitor the autophagic flux induced by Paxil, we transfected NSCLCL cells with GFP-mCherry-LC3 tandem plasmids (Figure 5A). HBSS treatment was used as a starvation mimic and promoted autophagic flux to produce a dominant number of autolysosomes. The GFP-mCherry-LC3 fusion protein was able to mark the autolysosomes by showing red fluorescence since the green fluorescence was quenched in an acidic environment. In contrast, Baf treatment ultimately resulted in an elevated pH in the autolysosomes due to its inhibition of lysosomal acidification. The GFP-mCherry-LC3 localized in the aberrant autolysosomes displayed both green and red fluorescence (i.e., yellow puncta). Similar to Baf, Paxil treatment also induced an increase in yellow puncta, which suggested that Paxil may inhibit lysosomal acidification. The co-localization of GFP and mCherry fluorescence was significantly increased in Paxil- or Baf-treated cells compared to the starvation group (HBSS treatment), as analyzed by a Pearson’s correlation coefficient. These findings indicate that Paxil, similar to Baf, appeared to block the late stage of autophagic flux.

Of note, GFP-mCherry-LC3 can also label autophagosomes, in which green combined with red fluorescence manifests as yellow fluorescence. Therefore, the increase of yellow puncta in Paxil-induced NSCLC cells could also reflect an increase in autophagosomes due to a blockade of autophagosome-lysosome fusion. To exclude this possibility, NSCLC cells were transfected with GFP-LC3 as a marker of autophagosomes and subsequently treated with DMSO (vehicle), chloroquine (CQ), Rapa, or Paxil. The lysosomes were labeled by LysoBrite^TM^ red. In the vehicle-treated cells, signals for lysosomes (red) but not GFP-LC3 puncta, were present. Rapa strikingly induced the formation of GFP-LC3 puncta, a large number of which were also co-localized with the lysosomes. This observation was in agreement with the phenomena that Rapa promotes autophagy. Treatment with CQ, a well-known autophagy inhibitor, was used to interfere with autophagosome-lysosome fusion. CQ treatment induced the accumulation of GFP-LC3 puncta that only occasionally colocalized with the lysosomes. In contrast, although Paxil inhibited autophagy, Paxil did not affect the colocalization of GFP-LC3 puncta and lysosomes (Figure 5B). Moreover, we observed the intracellular structures by transmission electron microscopy and discovered many autolysosomes (formed by autophagosome and lysosome fusion) in the cells treated with Paxil compared with the control group (Figure 5C). These results suggest that Paxil was unlikely to interfere with autophagosome-lysosome fusion.

**FIGURE 6:**
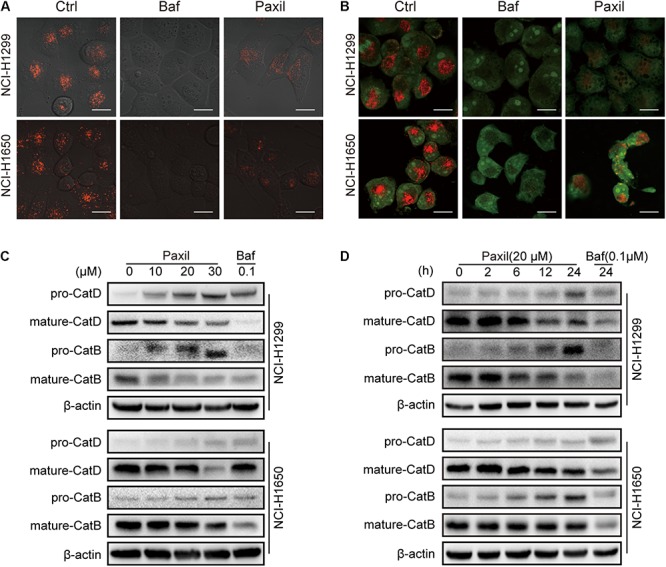
Paxil blocked autophagic flux by inhibiting lysosomal acidification. **(A,B)** Cells were treated with a vehicle, Baf (0.1 μM) or Paxil (20 μM) for 24 h and stained with lysosome-philic fluorescent dyes, including acridine orange (AO) and LysoTracker Red DND-99. The dyes are sensitive to acidity, and therefore, the fluorescence weakened with increased pH value. Images were acquired by a confocal laser scanning microscope. Scale bar: 20 μm. **(C,D)** Mature forms of cathepsins decreased with exposure to Paxil in a time- and dose-dependent manner.

Next, we investigated whether Paxil impaired autophagic flux via disrupting lysosomal acidification. LysoTracker and AO were applied, which emitted bright red fluorescence in the acidic vesicles. As expected, treatment with Baf, a well-known inhibitor of the lysosomal proton pump, completely abolished the LysoTracker (Figure 6A) and AO (Figure 6B)-related red fluorescence in both cell lines. To a lesser extent, treatment with Paxil for 6 h also significantly reduced the red fluorescence of both dyes compared to the vehicle control. Lysosomal cathepsins, such as cathepsin B (CatB) and cathepsin D (CatD), require an acidic environment for maturation. The ratio of mature to pro-forms of these cathepsins are widely used as indicators of an acidic lysosomal environment. As shown in Figures 6C,D, Paxil dose- and time-dependently reduced the mature forms of CatB and CatD in NCI-H1299 and NCI-H1650 cells, yet increased the levels of pro-CatB and pro-CatD. These data provide further evidence that Paxil may increase the pH of the acidic vesicles in NSCLC cells. Such effects, in turn, disrupted the processing of cathepsins in lysosomes, affected cargo degradation in the autolysosomes, and ultimately impaired autophagic flux.

### Paxil-Induced ROS Accumulation Promotes Mitochondria-Dependent Apoptosis via Activation of the MAPK Pathway

**FIGURE 7:**
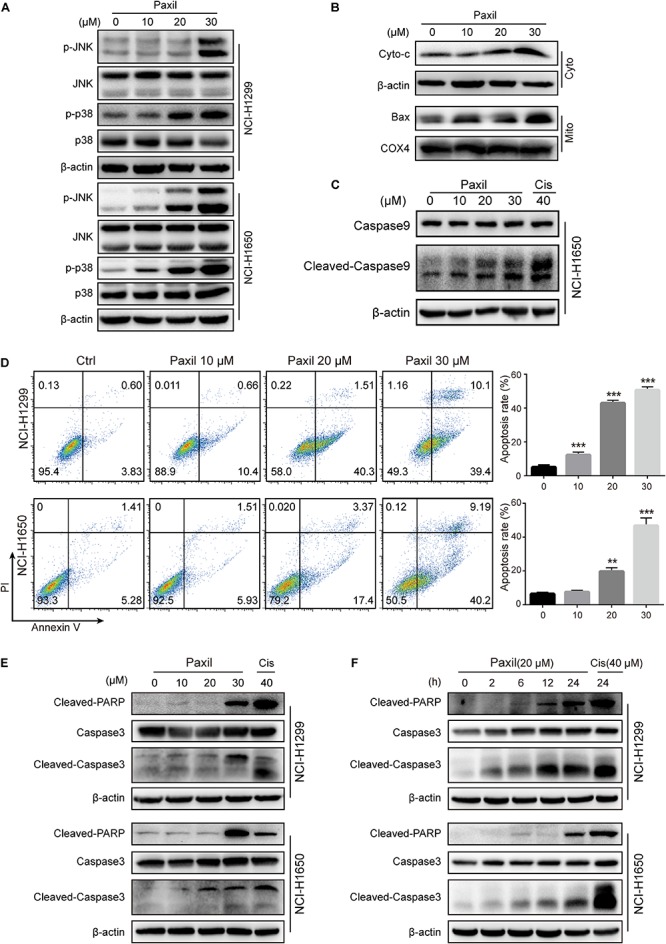
Paxil-induced ROS accumulation promoted mitochondria-dependent apoptosis by activating the MAPK pathway. **(A)** The upregulation of p-JNK and p-p38 induced by Paxil. **(B)** The increase of cytosolic cytochrome c and Bax localized in the mitochondria. Following Paxil treatment for 24 h, the cytosolic fractions and the mitochondrial parts in NCI-H1299 cells were separately extracted from the total lysates. The protein level in each fraction was examined by western blot. β-actin was used as an internal reference for the cytosolic fraction. COX4 was used as an internal reference for the mitochondrial fractions. Cyto-c, cytochrome c; Cyto, cytosolic fractions; Mito, mitochondrial fractions. **(C)** Paxil caused caspase 9 activation in NCI-H1650 human lung cancer cells. Following Paxil treatment for 24 h, the whole cell lysates were extracted and the protein level was examined by western blot. **(D)** The increased apoptosis rate of NSCLC cells treated with increasing concentrations of Paxil for 24 h. The drug-treated cells were stained with Annexin V/PI followed by the analysis of flow cytometry. **(E,F)** Paxil dose-dependently upregulated the level of cleaved-caspase 3 and cleaved-PARP in NSCLC cells.

Since Paxil induced the fragmentation of mitochondria and simultaneously blocked the clearance of damaged mitochondria, ROS accumulated in the cells (Figures 2B,C), which in turn was thought to activate the MAPK pathway. The immunoblotting results revealed that Paxil induced the upregulation of p-JNK and p-p38, the major kinases in the MAPK pathway, in a dose-dependent manner (Figure 7A). MAPK is a well-known pathway that mediates the translocation of Bax, a pore-creating protein, from the cytoplasm to the mitochondria, which leads to mitochondrial outer membrane permeability (MOMP). To confirm this assumption, the mitochondria and cytosolic fractions were separated and analyzed by western blot (Figure 7B). After 24 h, Paxil dose-dependently induced the re-distribution of Bax to mitochondria fractions (Mito) and the accumulation of Cyto-c in the cytosolic fractions (Cyto). The occurrence of MOMP was typically accompanied by the loss of the mitochondrial membrane potential which had been confirmed in Figure 2B.

The release of cytochrome c is a crucial factor that triggers the caspase cascade leading to programed cell death, so we then confirmed that Paxil caused caspase 9 activation by western blot (Figure 7C). Thus, an Annexin V/PI staining assay was performed to determine the rate of apoptosis following Paxil treatment. Compared to vehicle control, different doses of Paxil significantly increased the apoptotic rates in both NSCLC cell lines (Figure 7D). It was found that as many as 50% of cancer cells underwent apoptosis following Paxil (30 μM) treatment for 24 h. Apoptotic markers were then assessed by a western blot. Compared to the vehicle control, the levels of cleaved-caspase 3 and cleaved-PARP protein were upregulated by Paxil treatment in a dose- and time-dependent manner (Figures 7E,F). These results suggest that Paxil can induce apoptosis in NSCLC cells.

### Paxil Induces Apoptosis Through ROS-MAPK Signaling Pathways

**FIGURE 8:**
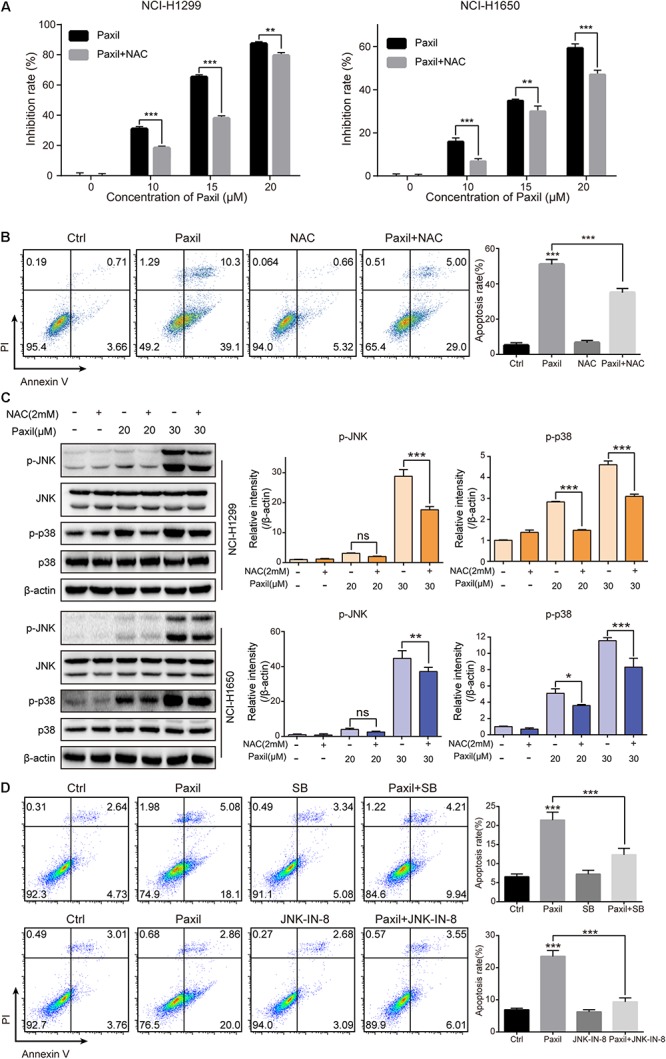
Paxil induced apoptosis by activating ROS-MAPK pathways. **(A)** Cells were treated with different concentrations of Paxil with or without NAC (2 mM) for 24 h, and the cell viability was determined by a CCK8 assay. **(B)** NCI-H1299 cells were treated with Paxil (20 μM) and NAC (2 mM) alone or in combination for 24 h and stained with Annexin V/PI for apoptosis analysis. **(C)** Cells were subjected to drug treatment as described in **(B)**. The protein level was examined by western blot. **(D)** NCI-H1299 cells were treated with Paxil (20 μM), SB (SB203580, a p38 inhibitor, 10 μM) and JNK-IN-8 (a JNK inhibitor, 5 μM) alone or their combination for 24 h, and then stained with Annexin V/PI for apoptosis analysis. ns, no significance; ^∗^*p* < 0.05; ^∗∗^*p* < 0.01; ^∗∗∗^*p* < 0.001.

To examine whether ROS plays an essential role in the Paxil-mediated cell response, we pre-treated cells with NAC, a ROS scavenger, followed by Paxil treatment. A CCK8 assay demonstrated that pre-treatment with NAC (2 mM) significantly reversed the cell growth-inhibiting activity of different doses of Paxil (Figure 8A). Coherently, NAC significantly suppressed Paxil-induced cancer cell apoptosis in an Annexin V/PI assay (Figure 8B; 34.0% vs. 49.4%, Paxil + NAC vs. Paxil, respectively). As expected, activation of the JNK and p38MAPK pathways by Paxil (20 or 30 μM) could also be attenuated in the presence of NAC (Figure 8C). To further confirm the essential roles of JNK and p38, we blocked the functions of JNK and p38 using JNK-IN-8 and SB203580, respectively. As evidenced in Figure 8D, the intervention of inhibitors to a great extent reversed Paxil-induced apoptosis, demonstrating JNK and p38 were indeed involved in the Paxil-induced anti-cancer effect. We also have examined ERK and p-ERK by western blot assay and p-ERK showed a dose-dependent upregulation after Paxil treatment (Supplementary Figure S3A). To verify whether p-ERK mediated the Paxil-induced apoptosis, we blocked the function of MEK1/2, the upstream kinase of ERK, using specific inhibitor (U0126-EtOH). As shown in Supplementary Figure S3B, with the addition of inhibitor, the level of p-ERK was reversed in a large part. However, the apoptosis rate of lung cancer cells was not rescued, or even worsened, suggesting ERK might not be involved in the Paxil-induced apoptosis (Supplementary Figure S3C).

### Paxil Inhibits Tumor Growth in a NCI-H1299 Tumor Xenograft Model

**FIGURE 9:**
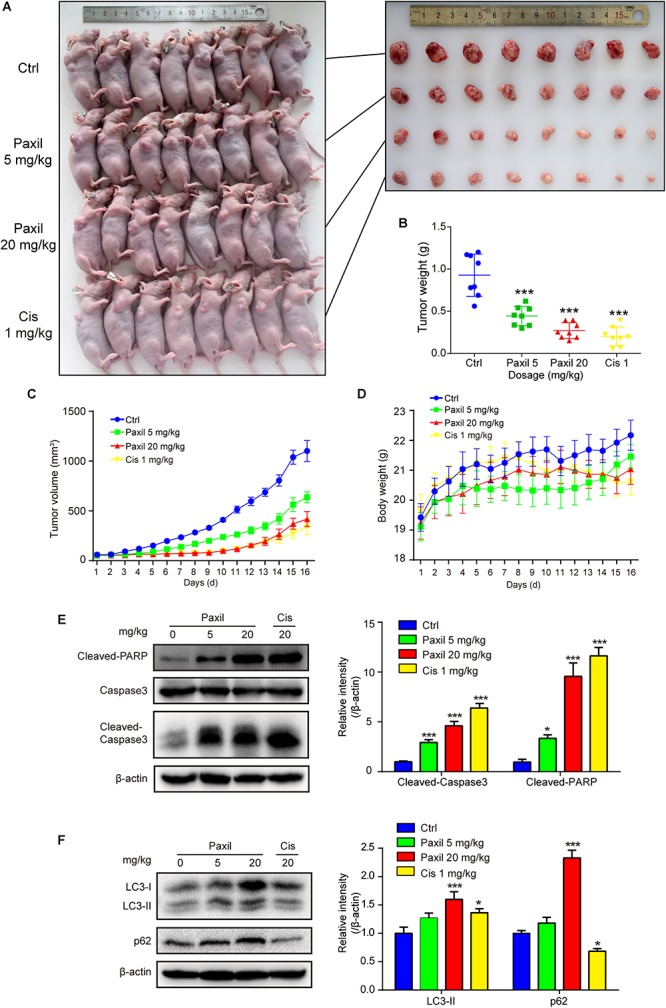
Paxil inhibited tumor growth in NCI-H1299 tumor xenograft models. **(A)** The anticancer-like activity of Paxil was evaluated in nude mice with xenografted tumors. A total of 32 mice were subcutaneously injected with NCI-H1299 cells, and then randomly assigned to four groups: (1) control (treated with normal saline); (2) Paxil-5 mg/kg; and (3); Paxil-20 mg/kg and cisplatin-1 mg/kg. **(B)** After 16 days, the mice were sacrificed for tumor tissue collection and the tumors were weighted. **(C)** The tumor volume was measured daily. **(D)** The mice weight was measured every day, and there was no significant decrease following drug treatment. Error bars represent the mean ± SE. **(E,F)** The autophagic and apoptotic states were measured in lung cancer xenografts by analyzing the indicated proteins in mice treated with either Paxil or a vehicle. The relative level of the indicated proteins was shown. Error bars represent the mean ± SD; one-way analysis of variance: ^∗^*p* < 0.05; ^∗∗∗^*p* < 0.001. Cis, cisplatin.

The anticancer-like activity of Paxil was evaluated in nude mice with xenografted tumors. A total of 32 mice were subcutaneously injected with NCI-H1299 cells, and then randomly assigned to four groups: (1) control (normal saline); (2) Paxil-5 mg/kg; (3) Paxil-20 mg/kg; and (4) Cisplatin-1 mg/kg. After 16 days, the mice were sacrificed for tumor tissue collection (Figure 9A). Treatment with Paxil significantly reduced the weight of the xenografted tumors compared to the vehicle (Figure 9B). Consistently, the average tumor volume was significantly smaller from Day 4 to the end of the study in the Paxil-20 mg/kg group compared with the control group (Figure 9C). The mice weight was measured every day, and there was no significant decrease following the drug treatment (Figure 9D). Since Paxil induced apoptosis and inhibited autophagic flux *in vitro*, we wondered whether Paxil exerted similar activity in the xenografted tumors *in vivo*. As demonstrated in Figure 9E, a dose of 20 mg/kg Paxil significantly increased the level of cleaved-caspase 3 and cleaved-PARP compared to the vehicle control. Furthermore, the analysis of autophagy markers demonstrated consistent results with the *in vitro* studies. Compared to the vehicle control, Paxil (20 mg/kg) significantly induced an accumulation of p62, and increased the ratio of LC3-II to β-actin in the tumor tissues (Figure 9F), suggesting that Paxil may also impair autophagic flux *in vivo*.

## Discussion

**FIGURE 10:**
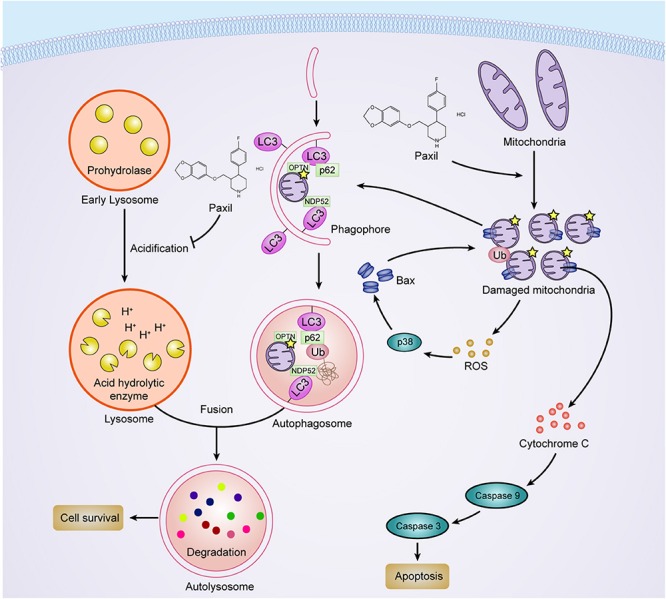
The proposed mechanisms of Paxil-inhibited autophagy and Paxil-induced apoptosis.

Paxil, a widely used antidepressant, showed anti-cancer activity in various types of cancer cells such as colon cancer cells and breast cancer cells (Cho et al., 2019; Jang et al., 2019). The major kinases of MAPK pathways such as p38, JNK was proposed to mediate the drug-induced apoptosis. However, the underly mechanism whereby paroxetine activates MAPK pathways remains to answer. In addition, although the priming of autophagy pathways dependent on FKBP51 was linked to the potency of paroxetine (Gassen et al., 2014), how Paxil perturbed the autophagic flux is still elusive. In this study, we discovered that Paxil exhibited tumor-suppressive functions as demonstrated by both *in vitro* and *in vivo* evidence in lung cancer cells, and two biological processes were revealed to be involved in the anti-tumor effect of Paxil (Figure 10): (1) Paxil directly targeted mitochondria and induced mitochondrial fragmentation via the overproduction of ROS; and (2) Paxil disrupted the autophagic degradation pathway by inhibiting lysosomal acidification. These dual actions consequently induced the accumulation of ROS, a detrimental factor for cells. Moreover, the accumulated ROS was able to activate the MAPK pathway and responded as a positive feedback loop to induce mitochondrial damage (Scherz-Shouval and Elazar, 2007). The increase of MOMP with the loss of mitochondria membrane potential is an initial step for subsequent apoptosis via the activated caspase cascade. Since cancer cells feature a large number of damaged mitochondria with the overproduction of ROS, a blockage of autophagy might result in a greater amount of ROS in cancer cells compared to normal cells (Simon et al., 2000; Vyas et al., 2016). Such metabolic differentiation may explain the prioritized killing effect of Paxil on cancer cells over normal cells.

Autophagy inhibition is considered to be a promising strategy for cancer therapy (Janku et al., 2011; Rubinsztein et al., 2012). Therefore, an increasing number of studies have focused on the identification of novel autophagy inhibitors; however, only a few inhibitors of autophagy (e.g., chloroquine and its derivative, hydroxychloroquine) are currently being evaluated in clinical trials aimed at assessing their efficacy (Mahalingam et al., 2014; Rangwala et al., 2014; Vogl et al., 2014). The main reason for this lack of clinical data is the unclear mechanism of the drug. In the present study, we demonstrated that Paxil blocked autophagic flux by inhibiting lysosomal acidification as follows: (1) Paxil inhibited the degradation of autophagosomes; (2) Paxil promoted substantial accumulation of autophagy substrates, such as LC3-II and p62; (3) Paxil quenched the fluorescence of lysosome-detecting probes (e.g., LysoTracker); and (4) Paxil attenuated the maturation of acid-sensitive lysosomal hydrolases (e.g., cathepsin B and D). Collectively, these findings indicate that Paxil interfered with the late stage of autophagic flux. Of note, since Paxil directly promoted mitochondrial damage, the initiation step of autophagy might be activated in cancer cells to promote survival. Compared with the vehicle, GFP-LC3 puncta were significantly induced and co-localized with mitochondria (red) following Paxil treatment (Figure 3F). This data demonstrates that mitophagy-relevant compartments were formed and engulfed the damaged mitochondria following Paxil treatment. Therefore, it is possible that mitophagy is initialized but arrested at the clearance stage. As such, the increased number of autophagosomes induced by Paxil might result from both the upregulation of the formation of autophagosomes and the inhibition of the downstream autophagic flux. Intracellular overloaded autophagosomes are per ser stress for cell survival. Several studies have reported that the accumulation of autophagosomes can result in tumor cell death, termed “autophagic cell death” (Zhou et al., 2015; Feng et al., 2018). However, while we used 3-MA to inhibit autophagosome formation, it failed to attenuate Paxil-induced cell death (Supplementary Figure S4). Thus, the Paxil-induced cell death cannot be simply classified as “autophagic cell death.” Since autophagy inhibition is considered as a promising strategy to synergize cancer chemotherapy (White, 2012), we also detected the killing effect of the combination of Paxil and cisplatin on NCI-H1299 cells. The result showed that the Paxil plus cisplatin combination treatment had a significantly increased rate of apoptosis as compared with cisplatin alone (Supplementary Figure S5).

When the autophagy pathway is obstructed, intracellular metabolic waste accumulates within the cell, and eventually disrupts cellular homeostasis (Yu et al., 2018). One typical source of cellular damage is ROS, an aging-promoting factor, which can also promote cellular apoptosis. Several studies have proposed that excessive ROS accumulation can activate the MAPK pathway and ultimately result in cell death. For example, palmitate was shown to induce H9c2 cell death through the activation of the ROS/MAPK signaling pathway (Liu et al., 2015). In addition, Isoliensinine was found to induce apoptosis in human breast cancer cells through ROS generation and p38/JNK activation (Zhang et al., 2015). Consistent with these findings, our present study demonstrates that the classical ROS-MAPK pathway mediates Paxil-induced apoptosis. We also determined that apoptosis induced by Paxil was mitochondria-dependent, based on the increase in MOMP followed by the release of cytochrome c and loss of membrane potential. However, it remains unknown which downstream effector of the MAPK pathway functions increase MOMP. Bax is a candidate since it can translocate to the mitochondria and create a pore if the guarding protein, BCL-2, becomes dysfunctional (Martinou and Youle, 2011). Moreover, there are several reports on the regulation of BCL-2 and Bax by MAPK. For example, Bax phosphorylation by JNK and p38 MAPK has been reported to initiate its activation and mitochondrial translocation (Kim et al., 2006). The JNK pathway also regulates mitochondrial apoptotic cell death via the modulation of Bcl-2 and Bax protein expression (Yamamoto et al., 1999; Schroeter et al., 2003; Asakura et al., 2008; Lee et al., 2008). As expected, our data indicate that treatment with Paxil induced the translocation of Bax to the mitochondria and the release of cytochrome c from mitochondria. To determine whether ROS plays an essential role in this process, we conducted a reverse assay in which ROS was neutralized by NAC, a ROS scavenger. The results showed the apoptosis was rescued and the key MAPK kinases (e.g., JNK and p38) were also deactivated. Collectively, our findings indicate that Paxil induces apoptosis in a mitochondria-dependent manner via the ROS-MAPK pathway.

## Materials and Methods

### Chemicals and Antibodies

Paroxetine hydrochloride (Paxil, T1636) was purchased from TargetMol (Shanghai, China). DMEM, streptomycin, penicillin, HBSS, FBS, Prolong Diamond DAPI (P36966) were ordered from Thermo Fisher Scientific (Waltham, MA, United States). Bafilomycin A1 (Baf, B1793) were obtained from Sigma Biotechnology (St. Louis, MO, United States). U0126-EtOH (U0126, S1102) and 3-Methyladenine (3-MA, S2767) were obtained from Selleckchem (Houston, TX, United States). CCK-8 reagent (CK04) was obtained from Dojindo Laboratories, Kumamoto, Japan. A BCA protein assay kit (MA0082) was from Meilunbio (Dalian, China). FITC Annexin V Apoptosis Detection Kit (556547) was from BD Pharmingen (San Jose, CA, United States). The anti-p62 antibody (5114), anti-PARP antibody (9542), anti-LC3B antibody (12994S), anti-P-JNK antibody (9251), anti-JNK antibody (9252), anti-p-Ub antibody (62802), anti-PINK1 antibody (6946), anti-Parkin antibody (4211), anti-COX4 antibody (4850), anti-NDP52 antibody (60732), anti-Optineurin antibody (58981), anti-ERK antibody (9102), anti-p-ERK antibody (9101), anti-Cytochrome C antibody (11940), anti-Caspase 9 antibody (9502), cleaved-caspase 3 antibody (9662) and MitoTracker Red CMXRos (9082) were from Cell Signaling Technology (Boston, MA, United States). The anti-CatD antibody (sc-13985), anti-Optineurin antibody (sc-166576) and anti-NDP52 antibody (sc-376540) was obtained from Santa Cruz Biotechnology (Dallas, TX, United States). The siRNAs were synthesized by RiboBio (Guangzhou, China). Peroxidase-labeled antibody to rabbit IgG (AS014) and peroxidase-labeled antibody to mouse IgG (AS003) was from ABclonal (Wuhan, China).

### Cell Culture

The human NSCLC cell lines, NCI-H1299 (ATCC^®^ CRL-5803^TM^) and NCI-H1650 (ATCC^®^ CRL-5883^TM^), were purchased from American Type Culture Collection (Rockville, MD, United States). All the cells were cultured in Dulbecco’s modified Eagle’s medium (DMEM) supplemented with 10% fetal bovine serum (FBS) and 100 U/mL penicillin/streptomycin at 37°C in a humidified atmosphere with 5% CO_2_.

### Cell Viability Assay

Cells were seeded onto 96-well plates at a density of 5 × 10^3^ cells/well and treated with different compounds for 24 h. The medium was removed, and 100 μL CCK-8 reagent (10 times dilution with DMEM) was added into each well. The plates were incubated for 2 h at 37°C then measured at 450 nm. Cell viability in each well was assessed by comparing the readouts of vehicle-treated cells.

### Colony Formation Assay

Cells were seeded into six-well plates at a density of 3000 cells/well and treated with Paxil at different concentrations. After 24 h, the medium was replaced with fresh complete DMEM. The cells were maintained for another seven days to allow the formation of colonies. Cell colonies were washed with PBS and fixed in 4% paraformaldehyde for 20 min. The colonies were stained with 0.01% (w/v) crystal violet in H_2_O for 10 min, following by thoroughly wash with H_2_O and air dried. The colonies were shot and counted by a researcher who was blinded for grouping information.

### CFDA-SE Cell Tracer Assay

Cells were labeled with CFDA-SE (C0051, Beyotime, Shanghai, China) and seeded into six-well plates overnight. The cells were treated with different doses of Paxil. After 24 h, the cells were trypsinized, washed with PBS, and resuspended in HBSS. The fluorescence intensity of the cells was then measured on a BD Accuri^TM^ C6 flow cytometer (BD Pharmingen, San Diego, CA, United States).

### 5-Ethynyl-20-deoxyuridine (EdU) Staining

Cells were seeded into a confocal dish at a density of 3 × 10^6^ cells/dish and treated with Paxil at different concentrations. After 24 h, the cells were administrated with EdU agent (C0071S, Beyotime, Shanghai, China) and cultured for an additional 8 h. The cells were then fixed in 4% paraformaldehyde for 20 min and washed with 3% BSA in PBS three times, followed by incubation with 0.3% TritonX-100 in PBS for 15 min and three washes of 3% BSA in PBS. The cells were incubated with 5 μg/mL Hoechst 33342 at room temperature for 10 min, washed with PBS, and mounted with Prolong Diamond (P36966, Karlsruhe, Germany). Images were obtained using a confocal microscope (LSM 800, Carl Zeiss, Jena, Germany).

### Cell Apoptosis Assay

Cells were plated into six-well plates at a density of 3 × 10^5^ cells/well and treated with different doses of Paxil for 24 h. The cells were trypsinized, washed with PBS, and resuspended with 600 μL annexin V binding buffer. The cells were mixed with 3 μL Annexin-V-FITC and incubated for 20 min at 37°C in the dark, followed by a PI (5 μL/sample) incubation for another 5 min in the dark. A total of 10,000 cells from each sample were analyzed with the FL1 channel and FL3 channel with a flow cytometer (BD Accuri C6, BD Pharmingen, San Diego, CA, United States).

### Transmission Electron Microscopy (TEM)

NCI-H1299 cells were plated into 10 cm dishes at a density of 1.5 × 10^6^ cells/dish and treated with either a vehicle or Paxil. After 24 h, the cells were collected and fixed with 2.5% glutaraldehyde for 12 h and incubated with osmium tetraoxide for 2 h at 4°C the specimens were embedded with epoxy resin. Sections 100 nm-thick were prepared and stained using uranyl acetate and lead citrate. Sections were imaged with a transmission electron microscope (HT7700, Hitachi, Tokyo, Japan).

### Mitochondrial Mass Detection

The NCI-H1299 cells were seeded into a six-well plate at a cell density of 3 × 10^5^ cells/well. The cells were treated with either a vehicle or Paxil for 24 h prior to fixing the cells with 4% paraformaldehyde for 15 min. After permeabilization in 95% methanol for 30 min, the cells were incubated in the antibody dilution buffer containing an anti-COX4 primary antibody (1:1000) for 2 h at room temperature and washed with PBS. After labeling the cells with an Alexa 488-conjugated secondary antibody, the fluorescence intensity of each group was examined with the FL1 channel of the flow cytometer.

### Western Blot Analysis

The cells were lysed with 1 × loading buffer for protein extraction. The proteins were separated via 12% sodium dodecyl sulfate-polyacrylamide gel electrophoresis (SDS-PAGE) and transferred onto PVDF membranes. The membranes were blocked with 5% skim milk in TBST for 2 h and incubated with primary antibodies at 4°C overnight. The membrane was washed with TBST (0.05% Tween 20 in Tris-buffered saline) three times and incubated with the secondary antibodies (diluted 1:4000). The bands were visualized with enhanced chemiluminescence (ECL) using an ECL detection system. Then the band density was quantified using ImageJ software (US National Institutes of Health, Bethesda, MD, United States).

### Plasmid Transfection Assay

Cells were seeded onto coverslips and cultured in 12-well plates for 24 h. Then, mCherry-GFP-LC3B plasmids were transfected using Lipofectamine 3000 reagent according to the manufacturer’s instructions (Invitrogen, Carlsbad, CA, United States). After 6 h, the medium was replaced with complete DMEM and cultured for 24 h. The cells were then treated with different chemicals for 24 h, fixed with 4% paraformaldehyde for 20 min, and washed with PBS three times. The cells were stained with Hoechst 33342 (5 μg/mL) at room temperature for 10 min and mounted on a glass slide using Prolong Diamond. Images were obtained using a confocal microscope (LSM 800, Carl Zeiss, Jena, Germany).

### Small Interfering RNA Transfection

The sequence of small interfering RNA for FUNDC1, BNIP3 and Nix were referred to the published articles (Bacon et al., 2007; Kuribayashi et al., 2011; Liu et al., 2012). NCI-H1299 cells seeded onto 15 cm dishes were transfected with small interfering RNA using riboFECT^TM^ CP Transfection Kit (R10035.4, RiboBio, Guangzhou, China) and cultured for 24 h followed by Paxil treatment. The mitochondrial parts in NCI-H1299 cells were separately extracted from the total lysates and then subjected to western blot. COX4 was used as an internal reference for the mitochondrial fractions.

### Acridine Orange (AO) Staining

Cells were seeded onto glass slides in 12-well plates and treated with different chemicals for 24 h. The medium was removed and the cells were washed with PBS three times. The cells were stained with AO (5 μg/mL, A6014, Sigma, St. Louis, MO, United States) and incubated at 37°C with 5% CO_2_ for 20 min. The samples were washed with PBS three times before imaging capture under a laser confocal scanning microscope equipped with an argon laser (excitation wavelength: 488 nm) and a 63 × objective lens. AO produces red fluorescence (emission filter: 620 nm long pass) in the acidic vesicles, whereas green fluorescence (emission between 520 and 560 nm) is emitted in the nuclear and cytosol compartments. The red and green fluorescence ratios were analyzed using ImageJ software.

### Measurement of Mitochondrial Membrane Potential

Cells were seeded into six-well plates and treated with Paxil for 24 h. Cells were then harvested, washed with PBS, and stained with JC-1 (551302, BD Pharmingen, San Jose, CA, United States) for 15 min at 37°C in the dark. After staining, the cells were washed with PBS and suspended in HBSS. A total of 10,000 cells from each sample were recorded in the FITC and PE channels using a BD Fortessa flow cytometer.

### Immunofluorescence Assay

Cells were seeded onto glass coverslips in a 12-well plate and treated with different chemicals for 24 h. After treatment, cells were fixed with 4% paraformaldehyde (PFA) for twenty minutes then rinsed for three times with PBS for five minutes. Then cells were blocked in antibody dilution buffer containing 10% FBS and 0.3% Triton X-100 in PBS for two hours at room temperature. Then cells were incubated with primary antibodies (1:500 dilution) overnight at 4°C. Then cells were washed with PBS for three times and blotted with fluorescent anti-rabbit secondary antibody (1:500 dilution) for 1 h at room temperature. Cells were rinsed in PBS three times before mounting by ProLong Diamond Antifade mounting medium with DAPI. Cells were imaged on a ZEISS LSM800 confocal laser scanning microscopy platform.

### LysoTracker Red Staining

Cells were seeded onto glass slides in 12-well plates and treated with different chemicals for 24 h. Cells were then loaded with LysoTracker Red (50 nM, L7528, Thermo, Waltham, MA, United States). After 20 min, the cells were rinsed with PBS and observed under a laser confocal scanning microscope (excitation wavelength: 555 nm). Images were captured for analyzing the fluorescent intensity with ImageJ software.

### MitoTracker Red CMXRos Staining

Cells were seeded onto glass slides in 12-well plates and treated with different compounds for 24 h. Cells were then stained with MitoTracker Red CMXRos (200 nM). After 20 min, the cells were rinsed with PBS and observed under a laser confocal scanning microscope equipped with a 63 × objective lens and an argon laser (excitation wavelength: 555 nm). Images were captured for analyzing fluorescent intensity with ImageJ software.

### Mitochondrial and Cytosolic Fractionation

A mitochondrial protein extraction kit (BB-3171, BestBio, Shanghai, China) and cytosolic protein extraction kit (BB-3113, BestBio, Shanghai, China) were adopted to collect mitochondrial and cytosolic proteins, respectively. After treatment, the cells were washed with PBS and harvested. The cells were resuspended in 500 μL mitochondrial or cytoplasmic protein reagent and incubated on ice for 20 min with vertexing every 5 min. The mitochondrial protein was prepared by centrifugation at 11,000 × *g* for 20 min. The cytoplasmic protein was prepared by centrifugation at 16,000 × *g* for 5 min. The cytoplasmic and mitochondrial proteins were stored at −80°C until they were used in a western blot analysis.

### Measurement of Intracellular ROS

Cells were seeded into six-well plates and treated with different chemicals for 24 h. Cells were then harvested, washed with PBS, and stained with 10 μM DCFH-DA (D399, Thermo, Waltham, MA, United States) for 15 min at 37°C in the dark. After staining, the cells were washed with PBS and suspended in HBSS. A total of 10,000 cells from each sample were recorded in the FL1 channel using a BD Accuri C6 flow cytometer.

### Xenograft Tumor Model

Xenografts were established by subcutaneous inoculation of 5 × 10^6^ cells into the right flank of nude mice. The xenograft tumor size was measured with a Vernier scale every five days. The mice began to receive different treatments (i.e., vehicle, Paxil [5 mg/kg], Paxil [20 mg/kg]) via an intraperitoneal injection when the tumor volume reached 0.1 mm^3^. After 13 days, the mice were sacrificed with CO_2_. The tumor tissues were harvested for weighting tumors, protein isolation, and western blot analysis.

### Statistical Analysis

All experiments were repeated at least three times. Data were expressed as the mean ± standard deviation (SD). The statistical differences were evaluated using a one-way analysis of variance (ANOVA) followed by a *post hoc* test. *p* < 0.05 were considered statistically significant.

## Conclusion

Our present study demonstrates that Paxil, a commonly used anti-depressant drug, functioned as a potent inhibitor of autophagy in lung cancer cells. Through impairing the acidic environment in lysosomes, Paxil can arrest late-stage autophagic flux. In addition, Paxil can induce the fragmentation of mitochondria. This dual function consequently caused an accumulation of ROS, which in turn activated MAPK to promote apoptosis in lung cancer cells. This anti-cancer effect of Paxil was also verified in an *in vivo* xenograft mouse model.

## Data Availability Statement

All datasets generated for this study are included in the article/Supplementary Material.

## Ethics Statement

The experiments using nude mice were approved by the Animal Ethics Committee at Guangzhou University of Chinese Medicine.

## Author Contributions

JX and XL: conceptualization. KW, YJZ, and BC: methodology. TY, YJZ, and JK: software. QG and BD: validation. KW and HW: formal analysis. KW, QG, and TY: investigation. YJZ and YL: resources. QG, YL, and YLZ: data curation. KW: writing – original draft preparation. XL and JX: writing – review and editing. KW, YLZ, and JK: visualization. XL: supervision. JX: project administration and funding acquisition.

## Conflict of Interest

The authors declare that the research was conducted in the absence of any commercial or financial relationships that could be construed as a potential conflict of interest.
