# Supplementary material for: Blockage of Autophagic Flux and Induction of Mitochondria Fragmentation by Paroxetine Hydrochloride in Lung Cancer Cells Promotes Apoptosis via the ROS-MAPK Pathway
Source: Front Cell Dev Biol. 2020 Jan 22;7:397. doi: 10.3389/fcell.2019.00397 (PMC6987457; doi:10.3389/fcell.2019.00397)
Supplement: Supplementary file 1 [file Table_1.DOCX]

***Supplementary Materials***

**Supplementary Figures**


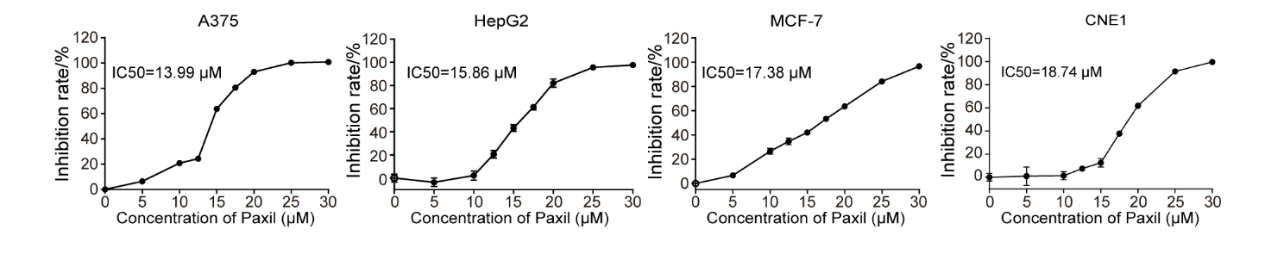


**Figure S1. Paxil inhibits the proliferation of multiple cancer cell lines.** A375, HepG2, MCF-7 and CNE1 cells were treated with a gradient concentration of Paxil for 24 h, and cell viability was determined by a CCK8 assay and visualized with GraphPad software. The median inhibitory concentration (IC50) was estimated by log (inhibitor) vs. normalized response nonlinear fit.


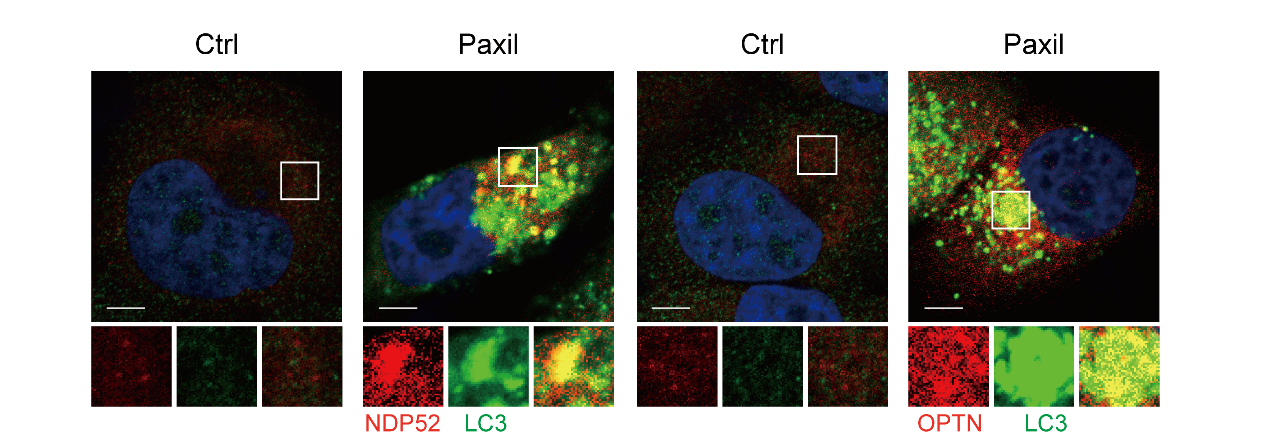


**Figure S2. Co-localization of NDP52 or OPTN and LC3.** Immunofluorescence staining assay was performed to detect the co-localization of NDP52 or OPTN and LC3. LC3 and NDP52/OPTN were labeled by green fluorescence and red fluorescence, respectively. Scale bar, 5 μm.


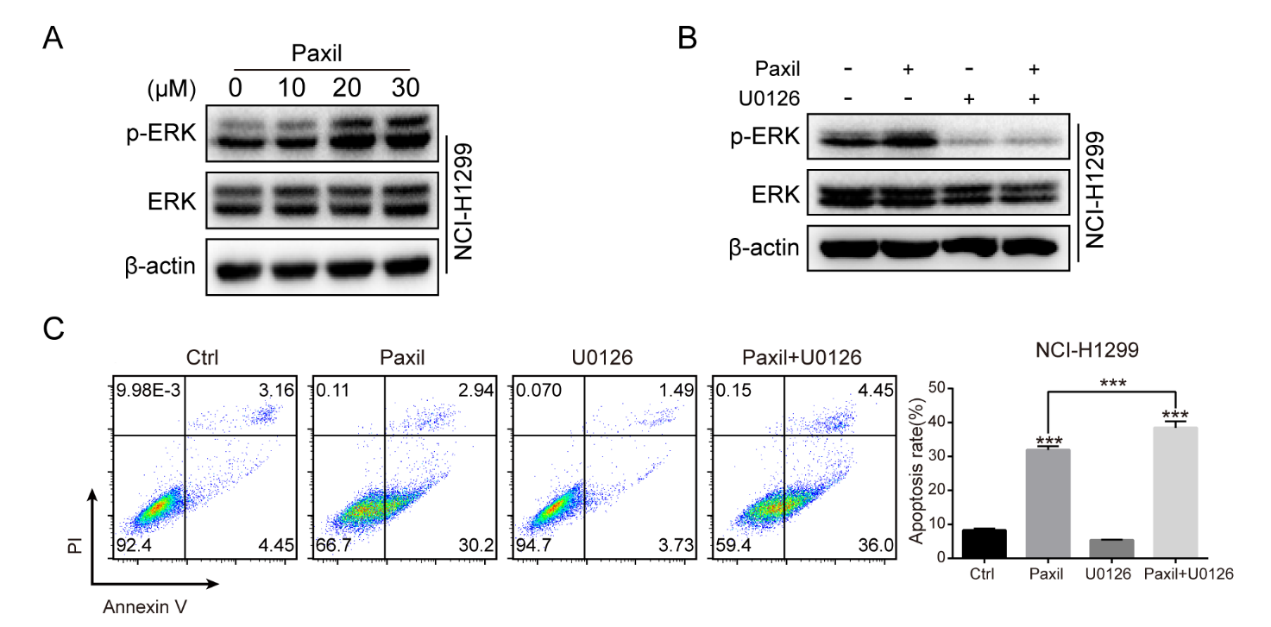


**Figure S3. Activation of ERK did not contribute to Paxil-induced apoptosis.** (**A**) Upregulation of p-ERK induced by Paxil. Cells were treated with various concentrations of Paxil for 24 h, then the indicated proteins were detected by western blot. (**B**) Inhibition of MEK1/2 reversed the Paxil-induced upregulation of p-ERK. Cells were treated with Paxil (20 μM) or U0126 (20 μM, a specific inhibitor of MEK1/2) or their combination for 24 h, respectively, followed by western blot assay. (**C**) The apoptosis percentage of NCI-H1299 cells analyzed by flow cytometry. Cells were treated with Paxil (20 μM) or U0126 (20 μM) or their combination for 24 h, respectively, followed by Annexin V/PI staining (left panel). The apoptosis rate in three independent flow cytometry experiments was quantified (right panel). Error bars represent the means ± S.D. of three independent experiments; ***p < 0.001.


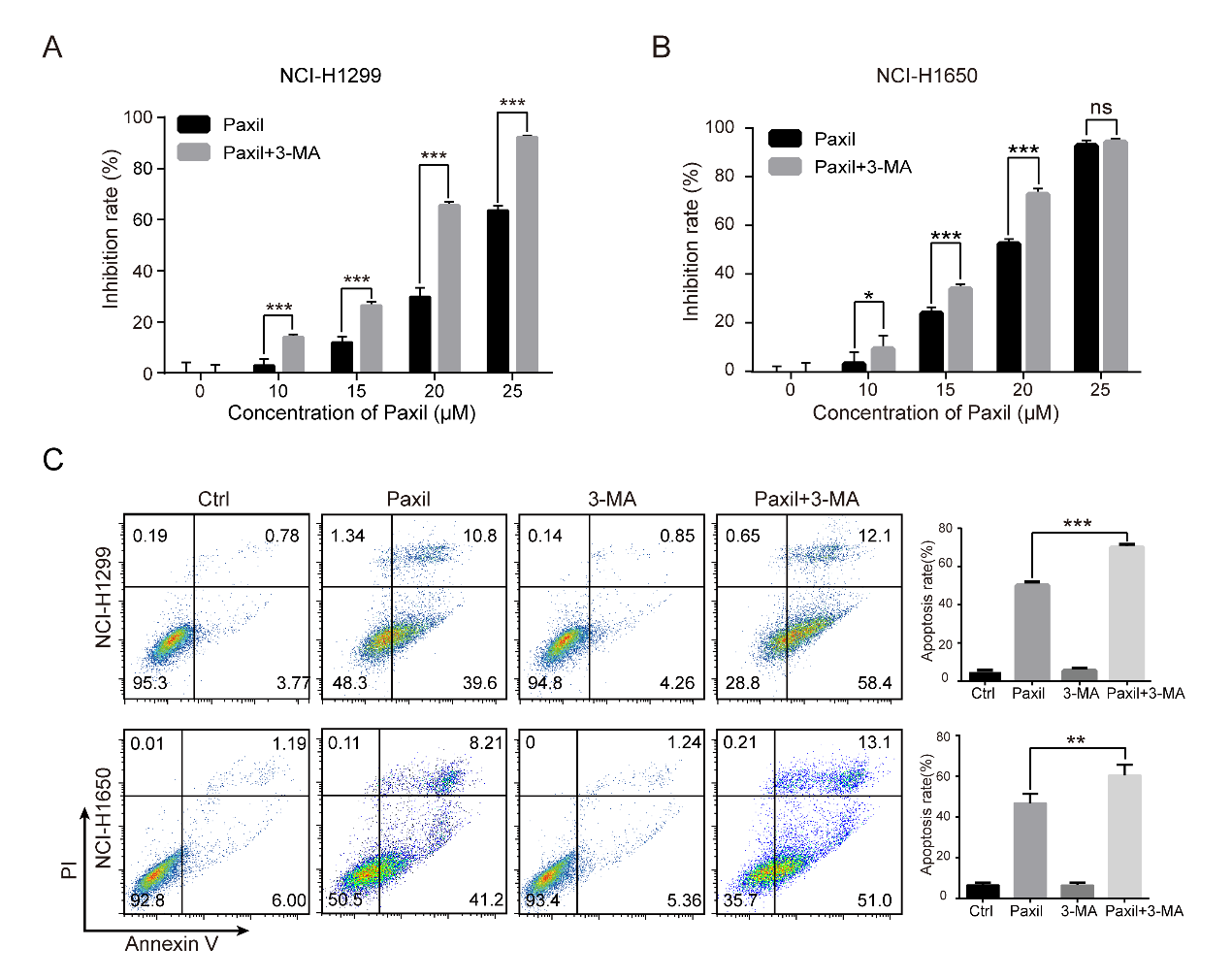


**Figure S4. Blocking autophagosome formation failed to protect cells against Paxil-induced cell death.** (**A, B**) Cell viability of NCI-H1299 (A) and NCI-H1650 cells (B) treated with a series of concentrations of Paxil or Paxil combined with 3-MA (5 mM) for 24 h. (**C**) The apoptosis percentage of NCI-H1299 and NCI-H1650 cells analyzed by flow cytometry. Cells were treated with Paxil (20 μM) or 3-MA (5 mM) or their combination for 24 h, respectively, followed by Annexin V/PI staining (left panel). The apoptosis rate in three independent flow cytometry experiments was quantified (right panel). Error bars represent the means ± S.D. of three independent experiments; *p < 0.05, **p < 0.01, ***p < 0.001.


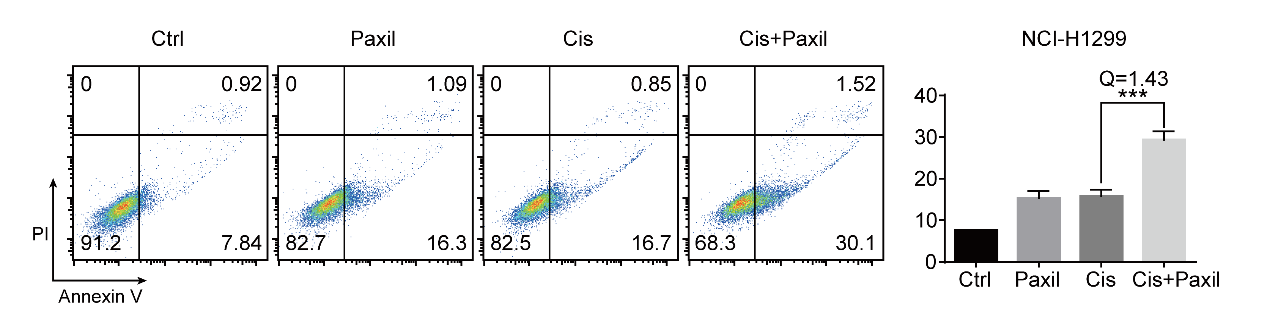


**Figure S5. Paxil plus cisplatin combination treatment had a significantly increased rate of apoptosis as compared with cisplatin alone.** NCI-H1299 cells were treated with various concentrations of Paxil for 24 h, followed by Annexin V/PI staining (left panel). The apoptosis rate in three independent flow cytometry experiments was quantified (right panel). Error bars represent the means ± S.D. of three independent experiments; ***p < 0.001. Q value = 1.43 > 1.15 represents a synergistic effect.
